# Cholera at Kurrachee

**Published:** 1846-12

**Authors:** 


*Cholera at Kurrachee.*—The seaport of Kurrachee, formerly one of the healthiest military stations, has, ever since it became known to us, been visited triennially with cholera. In 1839, and again in 1842, the amount of suffering occasioned by it was terrible, yet slight to that which it has just endured. Between the 13th and 23d June, above 8000 human beings were cut off by it, including 895 Europeans, of whom 815 were fighting men, 595 sepoys; and, it is believed, about 7000 natives, besides camp followers, and inhabitants of the town, have died. The pestilence had quitted Kurrachee, and was apparently creeping up the river. Fever of a very fatal kind had made its appearance amongst the European soldiers at Sukkur: its triennial visit is to be looked for next year. Her majesty's 17th had chiefly suffered: it was said they were to be moved down, while her majesty's 86th were to move up to Hydrabad.

An extremely dry season has been followed by an unusually wet one; between the 9th of June and the 17th July upwards of 35 inches of rain fell.

The Kurrachee Advertiser states that 7000 of the inhabitants have been cut off by cholera—nearly 9000 victims in ten days' time.

Of the 60th Rifles there died 4 sergeants, 101 men, 4 women, and 3 children. Total, 112.

Of the 86th Regiment: 1 officer, 24 sergeants, 329 men, 17 women, and 16 children. Total, 387.

Of the Company's Artillery: 4 sergeants, 19 men, 2 women, and 6 children. Total, 31.

Of the 1st Bombay European Regiment (Fusiliers): 1 officer, 18 sergeants, 314 men, 9 women, and 23 children. Total, 365.

Of the 3d N. I., 310 sepoys; of the 12th N. I., 236 sepoys; of the Belooch Batt., 49. Grand total, 1490; independent of the inhabitants.—*N. & M. Gaz.—Ibid.*

